# Delayed formation of pulmonary artery stump thrombus: a case report and review of the literature

**DOI:** 10.1186/1477-9560-7-7

**Published:** 2009-06-10

**Authors:** Monika Joshi, Umar Farooq, Sandeep Mehrok, Nadine Srouji

**Affiliations:** 1 Department of Medicine, PinnacleHealth System, Harrisburg, Pennsylvania, USA

## Abstract

Pulmonary artery stump thrombosis is a recognized complication after pneumonectomy. However, to our knowledge, there is only one case report of delayed development of this complication. We report the case of a 68 year-old man who presented with chest pain nearly ten years after undergoing a right pneumonectomy for lung cancer. Workup identified a pulmonary artery stump thrombosis. Due to the acute onset of his symptoms, the patient was anticoagulated, and his chest pain resolved. While the literature suggests that anticoagulation is not generally required for stump thromboses, we highlight features of this case that may indicate an increased risk of clinically important sequelae. Taking previous reports into account, we argue that a specific subset of patients with stump thrombosis may benefit from systemic anticoagulation.

## Background

The prevalence of pulmonary artery stump thrombosis after pneumonectomy is approximately 12%.[1,2]. Most authors regard such thromboses as benign entities because they are often discovered as an incidental finding on routine follow-up chest computed tomography (CT), and are rarely accompanied by pulmonary emboli. Accordingly, systemic anticoagulation therapy is not generally recommended. However, a recent case report linked a pulmonary artery stump thrombosis occurring ten years after right pneumonectomy with multiple pulmonary emboli and pulmonary hypertension.[3] These authors suggest consideration of prolonged postoperative anticoagulation in all patients undergoing right pneumonectomy to reduce the risk of thromboembolic events.

## Case presentation

A 68-year-old man presented to the emergency department (ED) complaining of intermittent pleuritic chest pain for one week. His medical history included non-small cell lung cancer, treated with right pneumonectomy and chemotherapy nearly ten years prior, chronic obstructive pulmonary disease (COPD), requiring home oxygen, hypertenstion, and prior tobacco use. Ten weeks before presenting to our hospital he was diagnosed with new onset paroxysmal atrial fibrillation. Warfarin was prescribed, which he did not take. Six weeks prior to admission he had been admitted for a COPD exacerbation, requiring intubation.

**Figure 1:**
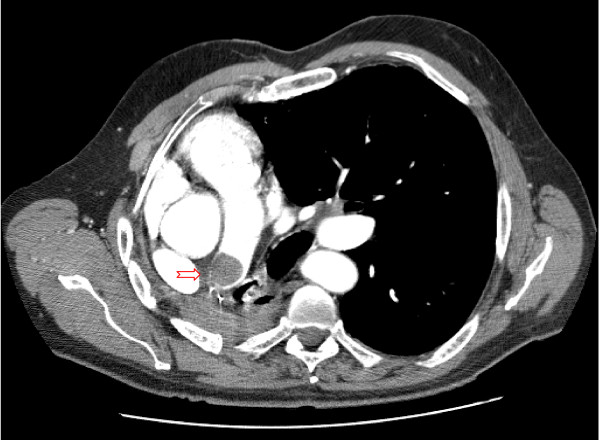
**Contrasted computed tomographic (CT) scan of the chest displaying a convex-shaped filling defect in the right pulmonary artery stump**.

Upon presentation to the ED, a contrasted chest CT was performed which revealed a large thrombus in the right pulmonary artery stump with no evidence of pulmonary emboli (Figure 1). Venous dopplers of his lower extremities did not reveal deep venous thrombosis. The patient had a chest CT with contrast done eighteen months earlier that did not show a pulmonary artery stump thrombus. He had also had two recent non-contrasted chest CT scans that were reviewed after the discovery of the right pulmonary artery thrombus. The routine scan performed three months earlier, for follow-up of his lung cancer, did not have any areas of increased density in the pulmonary artery stump, suggesting that there was no thrombus. However, review of the scan performed six weeks earlier, during his admission for COPD exacerbation, revealed an area of increased density in the right pulmonary artery stump, consistent with the location of the thrombus. These scans suggest that the thrombus developed during the period of clinical decline. An echocardiogram also performed at the time of the non-contrast chest CT, six weeks prior to his admission demonstrated a dilated right atrium with moderate right ventricle hypokinesis and elevated PA pressures of 40–45 mmHg consistent with mild pulmonary hypertension. The right ventricular hypokinesis was worse when compared to his previous echocardiogram two years prior. His echocardiograms were reported as being limited due to the dislocation of the heart within the chest cavity.

The patient was hemodynamically stable with a blood pressure of 120/82 mmHg and in atrial fibrillation at a rate of 83 beats per minute. He was admitted to a medical floor and anticoagulated with heparin and warfarin and his chest pain improved. In the following weeks he was hospitalized with an admission diagnosis of "COPD exacerbation" two more times. Despite the treatment goal of long-term anticoagulation, his INR was subtherapeutic at each of these admissions. A review of the patient's medical records from the four years prior to identification of the stump thrombus showed only a single previous admission for COPD exacerbation six weeks prior to his presentation to the ED.

## Discussion

The incidence and natural history of pulmonary artery thrombosis remain poorly elucidated. Controversy exists in the literature whether or not a right pulmonary artery stump is at greater risk of a thrombus than a left stump. Additionally, the role of anticoagulation remains unclear.

For anatomic reasons, the vascular stump is longer after a right pneumonectomy than after a left pneumonectomy.[4,5] Therefore, it has been postulated that stump thrombosis should occur more frequently on the right. Two retrospective studies reviewed chest CT scans of patients with a previous pneumonectomy. The incidence of pulmonary stump thrombus was 12% in both studies.[1,2] One study found a significantly higher incidence of right-sided thrombosis while the other found a nearly equal incidence of thrombus in the right and left stumps. Both studies identified significantly longer stumps among the patients who developed a thrombus, whether right or left pneumonectomy had been performed.

Only small numbers are available with which to estimate the incidence pulmonary embolism (PE) in patients with a documented post-pneumonectomy stump thrombosis. Kim et al. noted a PE in 1 of 18 such patients (5.6%); similarly, Kwek and Wittram found PE in 1 of 11 (9.1%). This incidence seems reassuringly low, especially since PE's are detected as an incidental finding on 4–5.7% of chest CT's performed on inpatients. [6-8] Kwek and Wittram note that five patients without PE at presentation went on to have a follow-up chest CT; in no case did thrombus propagate outside of the stump. They concluded that post-pneumonectomy artery stump thrombosis has a benign natural history. Of note, one of four patients in their series who did not have a follow-up chest CT reportedly died of pulmonary hypertension. It is conceivable that recurrent, undiagnosed PE's contributed to pulmonary hypertension in this case; however, the fact that this patient was chronically anticoagulated makes this scenario less likely. In Kim's study, 38% (5/18) of the patients had an increase in the size of the stump thrombus.

Kwek and Wittram introduced the concept of defining the shape of a stump thrombus as either convex or concave. Both patients in their series in whom new thrombi were identified, including the lone patient with PE and the patient with pulmonary hypertension, had convex thrombi. They also found that the maximal length of the convex thrombi was greater than the concave ones. Their findings suggest that convex thrombi are more acute and possibly less stable. It is important to note, though, that two of three patients with convex thrombi who had follow-up CT scans had either a resolution or a decrease in the size of their thrombus, even without anticoagulation.

Thomas et al. reported the case of a patient with a pulmonary artery stump thrombosis found ten years after right pneumonectomy with previously negative scans. Chronic microemboli were noted in the remaining lung, which led to pulmonary hypertension. This led the authors to advocate for chronic prophylactic anticoagulation. In our review of their patient's CT scan, the thrombus appears convex.

Likewise, our patient had a convex thrombus that developed many years after a right pneumonectomy and occurred during a period of decline in the patient's clinical condition. An echocardiogram done during his evaluation demonstrated moderate right hypokinesis that appeared worse compared to an earlier echocardiogram done. We postulate that the worsened right hypokinesis may have contributed to the development of his thrombus by causing low pulsatile flow, increasing stasis in the stump.

Our patient died two and a half months after the CT scan diagnosed a new stump thrombosis. On his last admission he was markedly hypoxic in the Emergency Department with an arterial blood oxygen pressure of 57 mmHg, despite the use of a 100% non-rebreather mask. Autopsy was not obtained, so the specific cause of his death remains unknown. We do know, however, that the patient had a convex pulmonary artery thrombus that was certainly not present on a previous contrast CT scan eighteen months prior and was not felt to be present on a non-contrast CT scan three months earlier. While non-contrasted CT scans are limited in their ability to reveal thrombi, we have a subsequent non-contrasted CT that shows an increased area of attenuation in the stump which was clearly not present on the scan done immediately before his hospitalizations. Clinically, his COPD, which had been stable, not requiring hospitalization for at least four years became significantly worse, requiring multiple hospitalizations and intubation. Based on this case, together with our review of the literature, we suggest that anticoagulation should be considered for convex stump thrombus or a new stump thrombus in the context of declining pulmonary status.

## Competing interests

The authors declare that they have no competing interests.

## Authors' contributions

MJ: Provision of care of patient, collection and assembly of data, review of the literature, manuscript writing; UF: collection of data; SM: provision of care of patient, review of the literature; NS: Collection and assembly of data, review of the literature, manuscript writing. All authors read and approved the final manuscript.

## Consent

The patient provided verbal consent to the authors to report his case and presentation. The patient expired prior to manuscript preparation and provision of written consent.
